# 3-(1*H*-Tetra­zol-5-yl)pyridinium chloride

**DOI:** 10.1107/S1600536809018972

**Published:** 2009-05-23

**Authors:** Jing Dai, Miao-Jia Yu

**Affiliations:** a Ordered Matter Science Research Center, College of Chemistry and Chemical Engineering, Southeast University, Nanjing 210096, People’s Republic of China

## Abstract

In the cation of the title compound, C_6_H_6_N_5_^+^·Cl^−^, the pyridinium and tetra­zole rings are nearly coplanar, making a dihedral angle of 5.05 (12)°. The cations and anions are connected by inter­molecular N—H⋯Cl hydrogen bonds, forming a centrosymmetric [2 + 2] aggregate. The aggregates are stacked along the *a* axis.

## Related literature

For applications of tetra­zole derivatives in coordination chemistry, see: Xiong *et al.* (2002 ▶); Wang *et al.* (2005 ▶). For the crystal structures of related compounds, see: Dai & Fu (2008 ▶); Wen (2008 ▶).
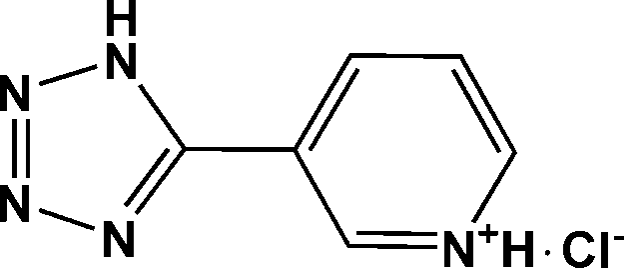


## Experimental

### 

#### Crystal data

C_6_H_6_N_5_^+^·Cl^−^
*M* *_r_* = 183.61
Monoclinic, 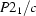

*a* = 4.2741 (9) Å
*b* = 8.1992 (16) Å
*c* = 23.559 (5) Å
β = 94.72 (3)°
*V* = 822.8 (3) Å^3^
*Z* = 4
Mo *K*α radiation
μ = 0.41 mm^−1^
*T* = 298 K
0.30 × 0.25 × 0.20 mm

#### Data collection

Rigaku Mercury2 diffractometer
Absorption correction: multi-scan (**CrystalClear**; Rigaku, 2005 ▶) *T*_min_ = 0.883, *T*_max_ = 0.921
8164 measured reflections
1862 independent reflections
1431 reflections with *I* > 2σ(*I*)
*R*_int_ = 0.041

#### Refinement

*R*[*F* ^2^ > 2σ(*F* ^2^)] = 0.040
*wR*(*F* ^2^) = 0.104
*S* = 1.03
1862 reflections
109 parameters
H-atom parameters constrained
Δρ_max_ = 0.22 e Å^−3^
Δρ_min_ = −0.28 e Å^−3^

### 

Data collection: *CrystalClear* (Rigaku, 2005 ▶); cell refinement: *CrystalClear*; data reduction: *CrystalClear*; program(s) used to solve structure: *SHELXS97* (Sheldrick, 2008 ▶); program(s) used to refine structure: *SHELXL97* (Sheldrick, 2008 ▶); molecular graphics: *SHELXTL* (Sheldrick, 2008 ▶); software used to prepare material for publication: *SHELXTL*.

## Supplementary Material

Crystal structure: contains datablocks I, global. DOI: 10.1107/S1600536809018972/is2410sup1.cif

Structure factors: contains datablocks I. DOI: 10.1107/S1600536809018972/is2410Isup2.hkl

Additional supplementary materials:  crystallographic information; 3D view; checkCIF report

## Figures and Tables

**Table 1:** Hydrogen-bond geometry (Å, °)

*D*—H⋯*A*	*D*—H	H⋯*A*	*D*⋯*A*	*D*—H⋯*A*
N1—H1*A*⋯Cl1^i^	0.86	2.25	3.0625 (18)	157
N2—H2⋯Cl1^ii^	0.86	2.23	3.0790 (18)	171

Symmetry codes: (i) 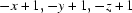; (ii) 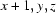.

**Fig. 1.**
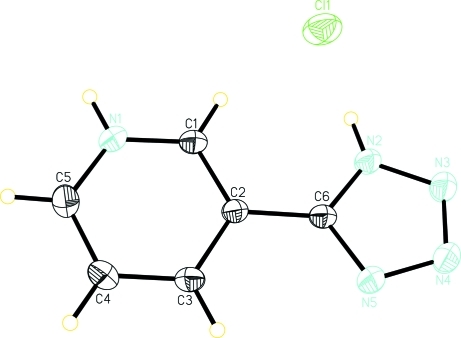
A view of the title compound with the atomic numbering scheme. Displacement ellipsoids were drawn at the 30% probability level.

**Fig. 2.**
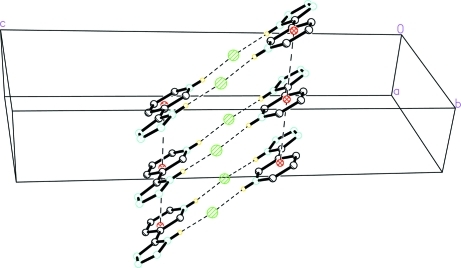
The crystal packing of the title compound, viewed approximately along the b axis showing the π–π and N—H···Cl interactions (dotted line) in the title compound. H atoms not involved in hydrogen bonding (dashed lines) have been omitted for clarity.

## supplementary crystallographic information

### Comment

In the past few years, more and more people have focused on the chemistry of tetrazole derivatives because of their multiple coordination modes as ligands to metal ions and for the construction of novel metal-organic frameworks (Wang *et al.*, 2005; Xiong *et al.*, 2002; Wen, 2008). We report here the crystal structure of the title compound, 3-(1*H*-tetrazol-5-yl)pyridinium chloride.

In the title compound, the pyridine N atom is protonated (Fig.1). The pyridinium and the tetrazole rings are nearly coplanar and only twisted from each other by a dihedral angle of 5.05 (12) °. The geometric parameters of the tetrazole rings are comparable to those in related molecules (Wang *et al.*, 2005; Dai & Fu, 2008).

The crystal packing is stabilized by aromatic π–π interactions between the benzene rings of the neighbouring cation systems. The *Cg*···*Cg*^iii^ distance is 4.274 (2) Å; *Cg* is the centroide of the C1—C6 benzene ring [symmetry code: (iii) *x* - 1, *y*, *z*]. The molecular packing is further stabilized by intermolecular N—H···Cl hydrogen bonds (Fig. 2 and Table 1).

### Experimental

Picolinonitrile (30 mmol), NaN_3_ (45 mmol), NH_4_Cl (33 mmol) and DMF (50 ml) were added in a flask under nitrogen atmosphere and the mixture stirred at 110°C for 20 h. The resulting solution was then poured into ice-water (100 ml), and a white solid was obtained after adding HCl (6 *M*) till pH=6. The precipitate was filtered and washed with distilled water. Colourless block-shaped crystals suitable for X-ray analysis were obtained from the crude product by slow evaporation of an ethanol/HCl (50:1 *v*/*v*) solution.

### Refinement

All H atoms attached to C and N atoms were fixed geometrically and treated as riding, with C—H = 0.93 Å (aromatic) and N—H = 0.86 Å, and with *U*_iso_(H) = 1.2*U*_eq_(C or N).

### Crystal data

**Table:**

C_6_H_6_N_5_^+^·Cl^−^	*F*(000) = 376
*M**_r_* = 183.61	*D*_x_ = 1.482 Mg m^−^^3^
Monoclinic, *P*2_1_/*c*	Mo *K*α radiation, λ = 0.71073 Å
Hall symbol: -P 2ybc	Cell parameters from 1862 reflections
*a* = 4.2741 (9) Å	θ = 3.0–27.3°
*b* = 8.1992 (16) Å	µ = 0.41 mm^−^^1^
*c* = 23.559 (5) Å	*T* = 298 K
β = 94.72 (3)°	Block, colorless
*V* = 822.8 (3) Å^3^	0.30 × 0.25 × 0.20 mm
*Z* = 4	

### Data collection

**Table:**

Rigaku Mercury2 diffractometer	1862 independent reflections
Radiation source: fine-focus sealed tube	1431 reflections with *I* > 2σ(*I*)
graphite	*R*_int_ = 0.041
Detector resolution: 13.6612 pixels mm^-1^	θ_max_ = 27.3°, θ_min_ = 3.0°
ω scans	*h* = −5→5
Absorption correction: multi-scan (*CrystalClear*; Rigaku, 2005)	*k* = −10→10
*T*_min_ = 0.883, *T*_max_ = 0.921	*l* = −30→30
8164 measured reflections	

### Refinement

**Table:**

Refinement on *F*^2^	Primary atom site location: structure-invariant direct methods
Least-squares matrix: full	Secondary atom site location: difference Fourier map
*R*[*F*^2^ > 2σ(*F*^2^)] = 0.040	Hydrogen site location: inferred from neighbouring sites
*wR*(*F*^2^) = 0.104	H-atom parameters constrained
*S* = 1.03	*w* = 1/[σ^2^(*F*_o_^2^) + (0.0463*P*)^2^ + 0.2136*P*] where *P* = (*F*_o_^2^ + 2*F*_c_^2^)/3
1862 reflections	(Δ/σ)_max_ < 0.001
109 parameters	Δρ_max_ = 0.22 e Å^−^^3^
0 restraints	Δρ_min_ = −0.28 e Å^−^^3^

### Special details

**Table:**

Geometry. All e.s.d.'s (except the e.s.d. in the dihedral angle between two l.s. planes) are estimated using the full covariance matrix. The cell e.s.d.'s are taken into account individually in the estimation of e.s.d.'s in distances, angles and torsion angles; correlations between e.s.d.'s in cell parameters are only used when they are defined by crystal symmetry. An approximate (isotropic) treatment of cell e.s.d.'s is used for estimating e.s.d.'s involving l.s. planes.
Refinement. Refinement of *F*^2^ against ALL reflections. The weighted *R*-factor *wR* and goodness of fit *S* are based on *F*^2^, conventional *R*-factors *R* are based on *F*, with *F* set to zero for negative *F*^2^. The threshold expression of *F*^2^ > σ(*F*^2^) is used only for calculating *R*-factors(gt) *etc*. and is not relevant to the choice of reflections for refinement. *R*-factors based on *F*^2^ are statistically about twice as large as those based on *F*, and *R*- factors based on ALL data will be even larger.

### Fractional atomic coordinates and isotropic or equivalent isotropic displacement parameters (Å^2^)

**Table:**

	*x*	*y*	*z*	*U*_iso_*/*U*_eq_	
Cl1	0.08068 (11)	0.24606 (6)	0.463087 (19)	0.04954 (18)	
N1	0.4537 (4)	0.72566 (17)	0.43139 (6)	0.0410 (4)	
H1A	0.5437	0.7521	0.4641	0.049*	
N2	0.6191 (4)	0.25335 (16)	0.35565 (6)	0.0389 (4)	
H2	0.7389	0.2624	0.3867	0.047*	
N3	0.5985 (4)	0.12105 (19)	0.32164 (7)	0.0467 (4)	
N4	0.3940 (4)	0.1567 (2)	0.27942 (7)	0.0495 (4)	
N5	0.2793 (4)	0.31072 (19)	0.28532 (7)	0.0446 (4)	
C1	0.5092 (4)	0.5771 (2)	0.41068 (7)	0.0365 (4)	
H1	0.6408	0.5050	0.4317	0.044*	
C2	0.3697 (4)	0.53090 (19)	0.35769 (6)	0.0307 (4)	
C3	0.1717 (4)	0.6434 (2)	0.32759 (7)	0.0373 (4)	
H3	0.0751	0.6155	0.2921	0.045*	
C4	0.1194 (5)	0.7958 (2)	0.35038 (8)	0.0444 (5)	
H4	−0.0111	0.8704	0.3304	0.053*	
C5	0.2639 (5)	0.8356 (2)	0.40346 (8)	0.0465 (5)	
H5	0.2301	0.9370	0.4196	0.056*	
C6	0.4224 (4)	0.3690 (2)	0.33340 (7)	0.0322 (4)	

### Atomic displacement parameters (Å^2^)

**Table:**

	*U*^11^	*U*^22^	*U*^33^	*U*^12^	*U*^13^	*U*^23^
Cl1	0.0494 (3)	0.0611 (3)	0.0360 (3)	0.0092 (2)	−0.0094 (2)	−0.0010 (2)
N1	0.0493 (9)	0.0431 (9)	0.0296 (8)	0.0010 (7)	−0.0028 (7)	−0.0053 (6)
N2	0.0425 (8)	0.0389 (8)	0.0337 (8)	0.0066 (6)	−0.0069 (6)	−0.0040 (6)
N3	0.0535 (9)	0.0397 (9)	0.0459 (9)	0.0048 (7)	−0.0019 (7)	−0.0063 (7)
N4	0.0576 (10)	0.0420 (9)	0.0468 (9)	0.0019 (7)	−0.0084 (8)	−0.0098 (7)
N5	0.0528 (9)	0.0392 (8)	0.0391 (8)	0.0018 (7)	−0.0121 (7)	−0.0045 (7)
C1	0.0395 (9)	0.0390 (9)	0.0298 (8)	0.0035 (7)	−0.0042 (7)	0.0021 (7)
C2	0.0321 (8)	0.0331 (8)	0.0265 (8)	−0.0003 (7)	0.0003 (6)	0.0011 (6)
C3	0.0392 (9)	0.0402 (9)	0.0311 (8)	0.0015 (7)	−0.0048 (7)	0.0028 (7)
C4	0.0473 (11)	0.0400 (10)	0.0450 (11)	0.0090 (8)	−0.0014 (9)	0.0073 (8)
C5	0.0560 (12)	0.0356 (10)	0.0482 (11)	0.0051 (9)	0.0066 (9)	−0.0025 (9)
C6	0.0319 (8)	0.0355 (9)	0.0286 (8)	0.0000 (7)	−0.0009 (6)	0.0030 (7)

### Geometric parameters (Å, °)

**Table:**

N1—C1	1.340 (2)	C1—C2	1.391 (2)
N1—C5	1.348 (2)	C1—H1	0.9300
N1—H1A	0.8600	C2—C3	1.404 (2)
N2—C6	1.345 (2)	C2—C6	1.470 (2)
N2—N3	1.347 (2)	C3—C4	1.385 (3)
N2—H2	0.8600	C3—H3	0.9300
N3—N4	1.302 (2)	C4—C5	1.387 (3)
N4—N5	1.366 (2)	C4—H4	0.9300
N5—C6	1.331 (2)	C5—H5	0.9300
			
C1—N1—C5	123.19 (15)	C2—C3—H3	119.8
C1—N1—H1A	118.4	N4—N3—N2	106.29 (14)
C5—N1—H1A	118.4	C3—C4—C5	119.18 (16)
N1—C1—C2	119.91 (15)	C3—C4—H4	120.4
N1—C1—H1	120.0	C5—C4—H4	120.4
C2—C1—H1	120.0	N3—N4—N5	110.72 (14)
C1—C2—C3	118.05 (16)	C6—N5—N4	105.96 (14)
C1—C2—C6	121.81 (14)	N1—C5—C4	119.22 (17)
C3—C2—C6	120.13 (14)	N1—C5—H5	120.4
C6—N2—N3	109.14 (14)	C4—C5—H5	120.4
C6—N2—H2	125.4	N5—C6—N2	107.90 (15)
N3—N2—H2	125.4	N5—C6—C2	125.52 (15)
C4—C3—C2	120.44 (16)	N2—C6—C2	126.58 (14)
C4—C3—H3	119.8		

### Hydrogen-bond geometry (Å, °)

**Table:**

*D*—H···*A*	*D*—H	H···*A*	*D*···*A*	*D*—H···*A*
N1—H1A···Cl1^i^	0.86	2.25	3.0625 (18)	157
N2—H2···Cl1^ii^	0.86	2.23	3.0790 (18)	171

Symmetry codes: (i) −*x*+1, −*y*+1, −*z*+1; (ii) *x*+1, *y*, *z*.
